# P-520. Barriers and facilitators towards HIV Pre-Exposure Prophylaxis (PrEP) prescription among healthcare providers in North Louisiana

**DOI:** 10.1093/ofid/ofae631.719

**Published:** 2025-01-29

**Authors:** Patricia Pichilingue Reto, Melanie Green, Alexandre Malek, Paulette Pinargote, Deborah Smith

**Affiliations:** Louisiana State University Health Sciences Center Shreveport, Shreveport, Louisiana; Louisiana State University Health Sciences Center Shreveport, Shreveport, Louisiana; Louisiana State University Health Sciences Center Shreveport, Shreveport, Louisiana; Lousiana State University Health Shreveport, Bossier City, Louisiana; Louisiana State University Health Sciences Center Shreveport, Shreveport, Louisiana

## Abstract

**Background:**

The HIV/AIDS epidemic remains a major global health challenge. To end the HIV epidemic, it is crucial to ensure that everyone has fair and equal access to HIV treatment and prevention, including PrEP. Identifying and addressing barriers to PrEP prescription to ensure equitable access to prevent new infections is essential to end the HIV epidemic. Therefore, this study aims to identify incentives and barriers towards HIV PrEP prescription among healthcare providers in North Louisiana.

**Figure 1.**
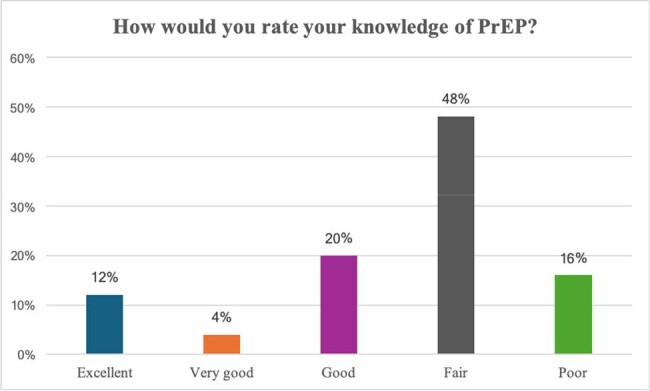
How would you rate your knowledge of PrEP? The majority of participants (48%) rated their knowledge about PrEP as fair, good in 20%, and it was reported as poor in 16% of participants.

**Methods:**

This is an ongoing observational cross-sectional survey study that aims to identify knowledge, attitudes, and intentions toward HIV PrEP among healthcare providers. Participants are faculty, residents, and fellows from the departments of Medicine, Pediatrics, Family Medicine and Obstetrics and Gynecology at an academic center in North Louisiana. A multi-modal recruitment strategy is being used to maximize the number of participants. We have conducted a preliminary descriptive analysis of the responses obtained from the study participants.

**Figure 2.**
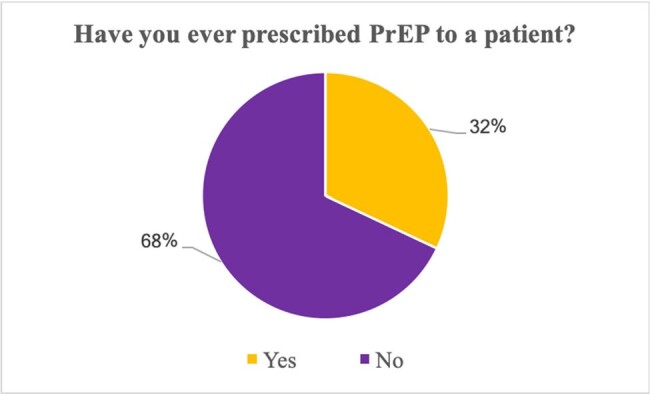
Have you ever prescribed PrEP to a patient? Among healthcare providers surveyed, the majority (68%) reported that they had never prescribed PrEP to a patient.

**Results:**

A total of 50 healthcare providers have taken the survey so far. Based on our initial findings, most study participants rated their knowledge of PrEP as either fair (48%) or poor (16%) (Fig.1). Concerning awareness of potential side effects, 44% reported poor knowledge. Most respondents (68%) had never prescribed PrEP (Fig.2). Barriers to PrEP prescription included: lack of PrEP provider training, lack of clinical guidelines for prescription and monitoring, challenges related to clinical and laboratory monitoring requirements, time constraints for patient education, and lack of insurance coverage (Fig.3). Facilitators to PrEP prescription included: access to resources such as PrEP prescription guidelines, implementation of Electronic Medical Records (EMR) order sets, clinical pharmacist support, and having peers who are knowledgeable and supportive of PrEP provision within their practice (Fig.4).

**Figure 3.**
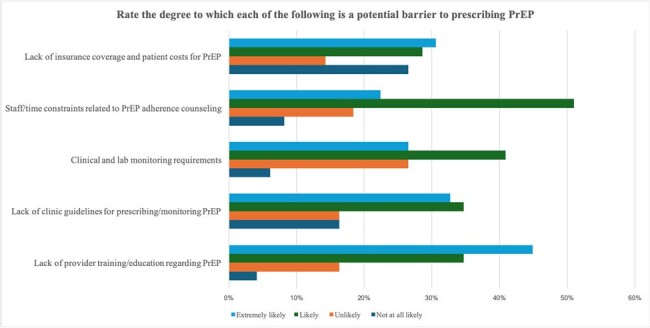
Potential barriers to prescribing PrEP. Lack of provider training on PrEP was considered a very likely barrier to prescribing PrEP by 45% of respondents, while more than 50% of participants considered staffing and time constraints likely to be a barrier.

**Conclusion:**

In order to make a significant contribution to HIV prevention, it is crucial to identify gaps in knowledge, attitudes, and practices among healthcare providers. This study’s ultimate goal is to improve PrEP implementation and dissemination, ultimately reducing new HIV infections among at-risk populations in Louisiana.

**Figure 4.**
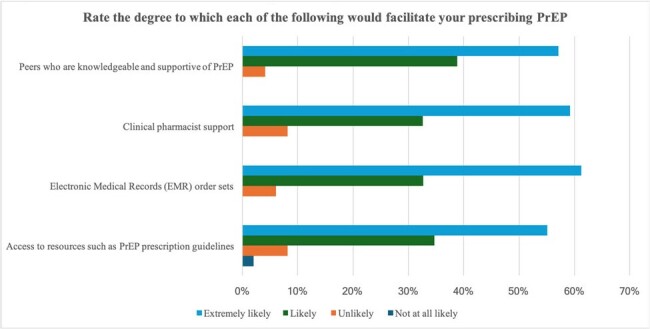
Facilitators of PrEP prescription. More than 60% of participants considered that having Electronic medical records (EMR) order sets was an extremely likely facilitator, followed by clinical pharmacy support and having knowledgeable and supportive peers of PrEP use.

**Disclosures:**

**All Authors**: No reported disclosures

